# Focal Muscle Vibration Combined with Electrical Stimulation for Post-Stroke Upper Limb Spasticity: A Pilot Study

**DOI:** 10.3390/bioengineering13101228

**Published:** 2026-10-21

**Authors:** Christopher L. Pulliam, Terri Z. Hisel, Sarah E. Grabinski, Victoria C. Whitehair, Jayme S. Knutson

**Affiliations:** 1 Department of Biomedical Engineering, Case Western Reserve University, Cleveland, OH 44106, USA; 2 Department of Physical Medicine and Rehabilitation, MetroHealth Rehabilitation Institute, Cleveland, OH 44109, USA; 3 Department of Population and Quantitative Health Sciences, Case Western Reserve University, Cleveland, OH 44106, USA; 4 Department of Physical Medicine and Rehabilitation, Case Western Reserve University, School of Medicine, Cleveland, OH 44106, USA

**Keywords:** electrical stimulation, hypertonia, spasticity, stroke, vibration

## Abstract

Post-stroke upper limb spasticity is a common condition characterized by increased muscle tone which interferes with rehabilitation, limits functional activities, and imposes a significant burden on patients and caregivers. Current treatments, including medications, injections, and physical therapy, often have limited effectiveness or produce side effects. Although some evidence supports focal muscle vibration (FMV) and electrical stimulation individually to reduce spasticity, the benefit of combining these modalities remains unclear. This pilot study explored the immediate effects of combining FMV with electrical stimulation on finger flexor spasticity in stroke survivors, aiming to identify combinations of FMV, neuromuscular electrical stimulation (NMES), and transcutaneous electrical nerve stimulation (TENS) that produce immediate reductions in hypertonia. Five stroke survivors were evaluated in repeated trials (804 trials in total) under 48 different conditions involving FMV (percussive and non-percussive), NMES (35 Hz and 100 Hz), and TENS, applied either alone or in combination to the spastic or antagonist muscles of the forearm. Interventions were applied for 10 s, and the Modified Ashworth Scale (MAS) score was assessed immediately before and after each stimulus. In a multinomial model adjusted for participant, percussive FMV with NMES, particularly at 100 Hz, yielded the greatest immediate reductions in MAS scores, with a higher likelihood of reducing MAS to 0 or 1 than percussive FMV alone; for the 100 Hz combination, this was observed in each of the five participants. In contrast, non-percussive FMV and TENS alone showed limited effectiveness, with a higher probability of no change. These preliminary findings indicate that combining percussive FMV with NMES can produce an immediate reduction in finger flexor hypertonia under the tested conditions. Confirmation in larger, blinded studies with longer stimulation and follow-up periods is needed to establish efficacy.

## 1. Introduction

Post-stroke upper limb spasticity is common, typically manifesting as tightness in the elbow, wrist, and/or finger and thumb flexor muscles (hypertonia). This condition limits range of motion and interferes with rehabilitation interventions. Consequently, it can also severely limit functional activities and self-care, lead to joint contractures, cause pain and fatigue, and impose significant caregiver, economic, and healthcare system burden [1,2]. Spasticity is defined as a motor disorder characterized by a velocity-dependent increase in tonic stretch reflexes and exaggerated tendon jerks [3,4]. Clinically, it is observed as resistance to joint movement felt when an examiner passively stretches a muscle by moving a limb or digit, and it generally increases if the stretching is performed quickly.

Post-stroke spasticity is believed to be caused by loss of supraspinal influence on baseline spinal interneuron inhibition, resulting in hyper-excitable muscle stretch reflexes [5]. Several spinal interneuron pathways are thought to play a role in the development of spasticity, including pathways involving reciprocal inhibition, presynaptic Ia inhibition, Ib nonreciprocal inhibition from Golgi tendon organs, and Renshaw cell inhibition [6,7]. Normally, supraspinal inputs control spinal reflexes by converging, along with primary peripheral afferents, on interneurons projecting to motor neurons. In particular, the reticular formation (RF) in the medulla is an inhibitory center that regulates muscle tone via the dorsal reticulospinal tract (dRST). Together with input from the dRST, descending corticospinal tracts control the balance of inhibitory and excitatory inputs on stretch reflex circuits, and thereby maintain normal muscle tone. The controlling drive on the RF is lost, however, in cortical and internal capsular strokes, so the facilitatory action of the medial RST runs unopposed, resulting in spastic hemiplegia [6].

It is estimated that 17% to 43% of stroke survivors develop spasticity [8,9,10], with upper limb spasticity being more prevalent and more severe than lower limb spasticity [11,12,13]. Treatment options include stretching, weight-bearing, splinting, and oral anti-spasticity medications (e.g., baclofen, dantrolene, or tizanidine) [14]. Each of these often have limited effectiveness, and the oral medications may cause side effects such as sedation or cognitive impairment. Neuromuscular injection therapy (e.g., botulinum toxin or phenol) is a widely used treatment option that is generally effective, although its effects can be variable from day to day and wear off after approximately 3 months, requiring re-injections that tend to lose effectiveness after several cycles. Implanted intrathecal baclofen pumps are occasionally used in more severely affected individuals, but this treatment lacks specificity [15], requires careful patient selection and close monitoring [16], a neurosurgical procedure, and percutaneous pump refilling every 6–12 weeks. There remains a need for simple and efficacious methods to reduce spasticity after stroke, ideally methods that patients can self-administer.

Focal muscle vibration (FMV) is the application of vibration to a specific muscle or its tendon using a mechanical vibration device. A systematic review of 8 randomized controlled trials of FMV for post-stroke upper limb spasticity found a positive effect of FMV in 6 of the 8 studies [17], but there is a need for systematic exploration of the relevant parameters (e.g., percussive vs. non-percussive FMV, application to spastic vs. antagonist muscle) to identify FMV parameters that have a higher likelihood of being efficacious. Electrical stimulation (ES) is another potential non-pharmacological modality to address post-stroke spasticity. Two forms of ES are available. Neuromuscular electrical stimulation (NMES) is delivered at intensities sufficient to recruit motor axons and therefore produces muscle contraction. Transcutaneous electrical nerve stimulation (TENS) is instead delivered at higher frequencies and lower intensities selected to activate sensory afferents without recruiting motor axons, and was originally developed as an analgesic intervention. The two modalities are therefore distinguished by their intended neural target, motor axons versus sensory afferents, rather than simply by the presence or absence of a visible contraction. NMES and TENS have each been studied for their effect on post-stroke spasticity [18,19], but not in combination with FMV.

FMV, NMES, and TENS each have potential to reduce muscle hypertonia by modulating spinal inhibition via activation of Ia and/or Ib afferent pathways and/or by altering the excitability of the corticospinal pathways via intracortical inhibition of the primary motor cortex [18,20,21,22,23,24,25]. The three modalities differ, however, in how they engage these pathways. FMV acts purely mechanically, deforming the muscle or tendon to activate primary spindle endings and Golgi tendon organs. NMES acts by two routes at once, directly depolarizing sensory afferents beneath the electrodes and mechanically loading spindles and tendon organs through the contraction it evokes. TENS, which does not evoke contraction, acts only through direct electrical activation of afferent fibers. Combining FMV with NMES therefore recruits a broader set of afferent inputs than either modality alone, which is one basis for expecting the combination to outperform either in isolation. Since each modality alone has potential to reduce spasticity, it is reasonable to speculate that simultaneous application with certain parameters could produce a more robust beneficial effect on muscle spasticity than either alone. Therefore, the purpose of this pilot study was to explore the effects of FMV and ES on finger flexor spasticity in stroke survivors with the goal of identifying stimulus parameters and combinations that produce immediate observable reductions in hypertonia after short bouts of application. The primary hypothesis was that combining percussive FMV with ES would produce larger immediate reductions in finger flexor hypertonia than percussive FMV alone; the primary outcome was the Modified Ashworth Scale (MAS) score of the finger flexors measured immediately after each 10-s stimulus. Because many stimulus conditions were screened, the study was designed as an exploratory, hypothesis-generating pilot rather than a confirmatory trial. Subsequent studies may then build on the findings of this pilot study by determining the effects of longer durations of the combinations that were found in this study to produce the largest immediate effect.

## 2. Materials and Methods

**Figure 1:**
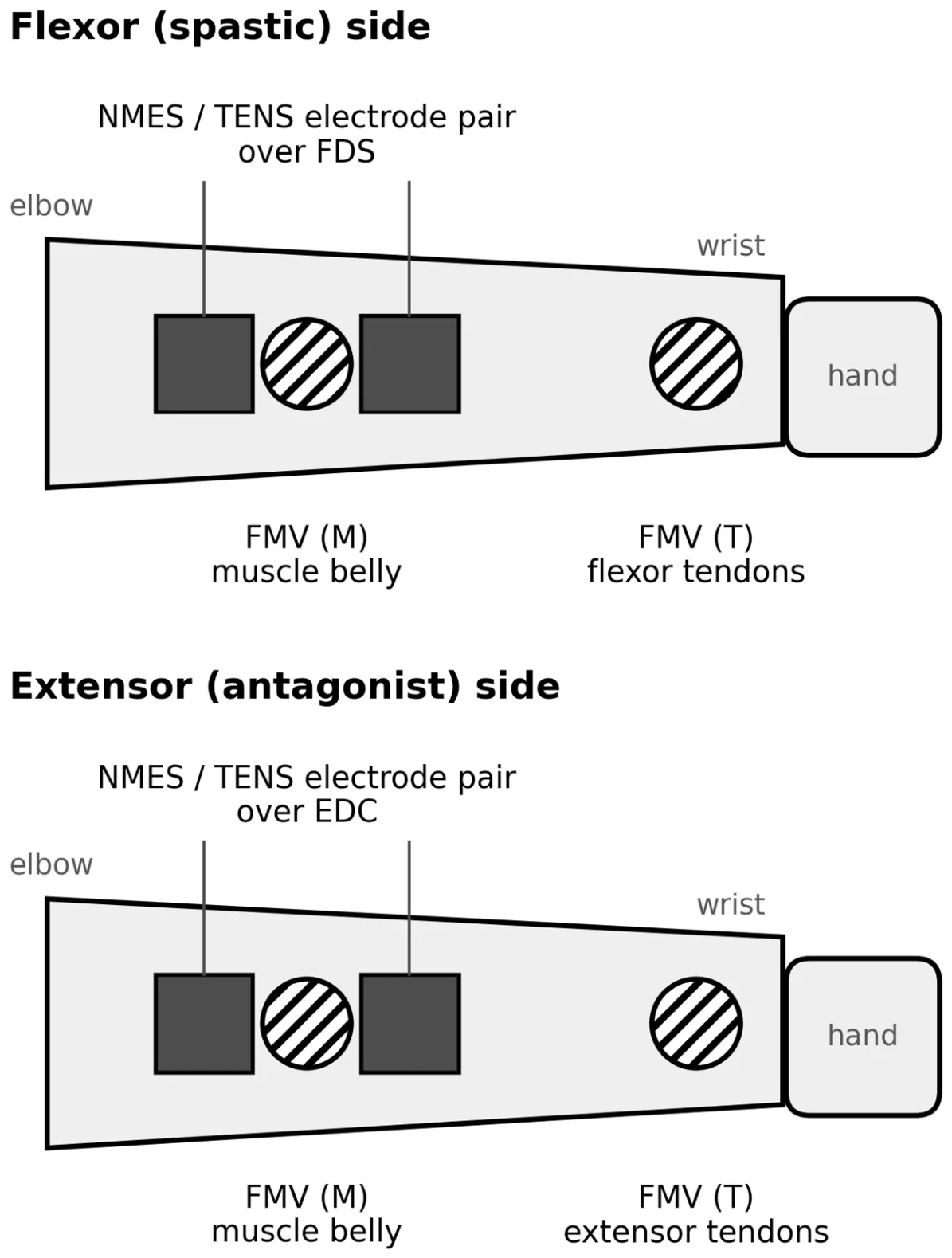
Stimulation sites on the paretic forearm (schematic, not to scale). On each side, NMES and TENS were delivered through one pair of 5 × 5 cm electrodes (dark squares) over the flexor digitorum superficialis (FDS) or the extensor digitorum communis (EDC). FMV (hatched circles) was applied either to the muscle belly (M), between the two electrodes, or to the tendons (T), just proximal to the wrist. Each test condition applied one stimulus, or two stimuli simultaneously, at these sites on the same or opposite sides of the forearm (Table 1).

This exploratory study was designed to identify stimuli that immediately reduce finger flexor hypertonia. The stimuli tested in this study included FMV, NMES, and TENS. They were applied alone and in some combinations with each other. There were two types of FMV—percussive and non-percussive, and two sites of FMV application—muscle belly or tendon. Percussive FMV and tendon application were selected with the possibility that the greater muscle stretch they produce could more effectively engage Ia and Ib afferent pathways, thereby enhancing inhibitory reflex pathways and promoting spasticity reduction through increased spinal reflex modulation. There were two types of NMES—35 Hz and 100 Hz. These frequencies were chosen to span the range used clinically: 35 Hz is typical of NMES for neuromuscular rehabilitation and produces a fused, comfortable contraction with relatively little fatigue, whereas 100 Hz matches the frequency used for TENS and for the non-percussive FMV device and lies within the 80–100 Hz range reported to be most effective for vibration-induced modulation of motor excitability [20,21,22]. Comparing the two also allowed us to examine whether a higher-frequency motor-level stimulus, which drives afferents at a higher rate, produced larger immediate effects. There were two sides of application for all stimuli—flexor (spastic) forearm and extensor (antagonist) forearm (Figure 1). Examining both spastic and antagonist muscle targeting was based on the effect of vibration in stimulating primary muscle spindle endings, activating Ia afferents that induce a reflex-mediated contraction in the vibrated muscle while inhibiting its antagonist [26]. When a combination of FMV and ES was tested, the two stimuli were either both applied to the same side of the forearm (flexor or extensor) simultaneously or to opposite sides of the forearm simultaneously. In all, 48 distinct test conditions were studied, plus a control condition in which no stimuli were applied (Table 1). For analysis, the 48 stimulus conditions were grouped into 11 treatment categories defined by the modality or pair of modalities applied (Section 2.5). The stimulus (or stimulus combination) was applied for 10 s, and finger flexor hypertonia was measured immediately prior to and after the stimulus. Details of the protocol and data analysis methods follow.

### 2.1. Participants

Stroke survivors with significant finger flexor hypertonia were enrolled, defined as having a MAS [27] score ≥2 for finger flexors. Additional selection criteria included >3 months from hemorrhagic or ischemic stroke; unilateral finger extensor paresis indicated by a score of ≤4 out of 5 on the manual muscle test (Medical Research Council scale); cognition and communication adequate to participate (investigator discretion); surface NMES of paretic finger extensors produced muscle contraction without pain; FMV of paretic finger extensors and flexors was well-tolerated; no intramuscular botulinum toxin injection of any upper limb muscle in the previous 3 months; no wrist or finger contractures; not insensate to touch on forearm or hand; no brainstem stroke; no concomitant neurologic diagnosis of peripheral nerve injury or any other upper motor disease or injury. No formal sample size calculation was performed. A convenience sample of five participants was considered appropriate for an exploratory pilot study in which each participant served as his or her own control across many within-participant conditions, the aim being to identify candidate stimulus combinations for subsequent, adequately powered studies rather than to test a confirmatory hypothesis.

The study was approved by the MetroHealth System Institutional Review Board (protocol number IRB21-00220). Written informed consent was obtained from all participants prior to any study procedures including those required to evaluate eligibility. Eligibility to participate was assessed via chart review, patient interview, and examination by the study principal investigator and study coordinator. After eligibility was confirmed, the Upper Extremity Fugl-Meyer (UEFM) assessment was administered as a measure of upper limb impairment [28] and baseline MAS scores of finger flexors were obtained.

### 2.2. Stimulation Devices

Two types of FMV devices were used—percussive and non-percussive. The percussive device (Percussion Massager distributed by FineLife Products, City of Industry, CA, USA) was shaped like a hair dryer and had a 1.25-inch (3.2 cm) diameter plastic flat disk-shaped applicator that pistoned in and out of the device at 60 Hz with a displacement (amplitude) of 5-mm. The percussive vibration produced a pulsing sensation on the skin. The non-percussive device (Hitachi Wand Massager distributed by Vibratex, Inc., Napa, CA, USA) was cylindrically shaped with a 2.25-inch (5.7 cm) rubber spherical applicator that vibrated in response to an internal offset unbalanced mass rotating at 100 Hz about a motor shaft. The non-percussive vibration produced a tingling/buzzing sensation on the skin; its displacement amplitude is not specified by the manufacturer and was not measured, but appeared considerably smaller than that of the percussive device. The head of each device was applied to the skin surface with a pressure adequate to make firm contact with the skin over the target muscle belly or its tendon for the duration of the trial. Muscle-belly (M) applications were made over the belly of the flexor digitorum superficialis (FDS) on the volar forearm or of the extensor digitorum communis (EDC) on the dorsal forearm, between the two electrodes of the NMES/TENS pair on that side, which were spaced just far enough apart to accommodate the applicator head. Tendon (T) applications were made over the corresponding flexor or extensor tendons on the distal forearm, approximately 2–4 cm proximal to the wrist crease (Figure 1).

NMES and TENS were applied through 2-inch (5 × 5 cm) square self-adhering surface (i.e., transcutaneous) electrodes (PALS Blue Neurostimulation Electrodes, Axelgaard Manufacturing Co., Ltd., Fallbrook, CA, USA). One pair of electrodes was positioned on the dorsal mid-forearm over the EDC to produce finger extension during NMES, and a second pair was positioned on the volar forearm over the FDS to produce finger flexion during NMES. When NMES and TENS were applied to the same side of the forearm, both were delivered simultaneously through the same electrode pair using a Y-adaptor, with one lead connected to the NMES output and the other to the TENS output. Photographs of the electrodes were taken during the first testing session so their positions could be replicated in subsequent sessions. The electrodes were connected to a custom-built multi-channel stimulator (UECU stimulator, Technical Development Laboratory, Cleveland FES Center, Cleveland, OH, USA) that was programmed to deliver biphasic symmetric rectangular current pulses. The pulse amplitude for both TENS and NMES was set at 40 mA. For TENS, the pulse frequency was 100 Hz and the stimulation intensity (i.e., pulse duration) was determined for each participant and pair of electrodes as that which produced the strongest tingling sensation without also producing a muscle contraction. For NMES, a pulse frequency of 35 Hz and 100 Hz were used. For each of those frequencies and each pair of electrodes a stimulation intensity (i.e., pulse duration) that produced a strong pain-free muscle contraction was determined. The resulting pulse durations were held constant for a given participant and electrode pair across sessions. Pulse durations ranged from 18 to 53 µs (TENS), 38 to 130 µs (NMES35), and 37 to 150 µs (NMES100). The stimulator was programmed to ramp the pulse duration from zero to those pre-determined pulse durations within one second after the NMES or TENS was turned on.

### 2.3. Experimental Protocol

The immediate effects on finger flexor spasticity of 10-s applications of FMV, ES, and several of their combinations were evaluated in each participant. Participants were seated in a large comfortable chair (Champion Manufacturing Inc., Ocala, FL, USA) with their paretic arm resting on the chair’s wide arm rest. Each trial followed the sequence illustrated in Table 2. During stimulation, the participant was instructed to relax the arm and not aid or resist any movement that may be produced during the application of stimulus [29]. When FMV only (without any ES) was being applied, a timer was used to count down 10 s; when NMES or TENS were being applied, the stimulator automatically shut off after 10 s.

The test order for the conditions listed in Table 1 was semi-random. Conditions were tested in groupings defined by the modality or pair of modalities involved (e.g., FMV_p_ + NMES35; Table 1). Within a session, the conditions belonging to one grouping were usually all presented, in an order chosen by the investigator at the time of testing, before moving on to another grouping (e.g., FMV_p_ only), and groupings were varied across sessions so that, for the four participants who completed all sessions, every grouping was tested in at least two sessions. The sequence was therefore participant-specific. Typically after every 8 trials the “control condition” was repeated, which was a trial in which no stimulus was applied but MAS was measured 10 s after the participant made and relaxed a fist. If the control condition showed no drop in MAS score, the stimulus trials resumed. If there was a drop in MAS score during the control condition, several minutes of rest was given, and the control condition was repeated. If repeated trials of the control condition continued to show reductions in MAS score (expected as muscle fatigues), the session was ended. The control trials thus served as an ongoing check for fatigue, habituation, and carry-over from preceding stimulation. Sessions typically lasted two hours. The goal was to repeat each of the 48 stimulus test conditions at least three times over multiple sessions per participant. Sessions were never conducted on consecutive days to avoid fatigue effects, and a single occupational therapist performed all MAS assessments. Adverse events, including pain, discomfort, fatigue, and skin reactions, were recorded when reported by participants or observed by the investigators.

### 2.4. Outcome Measures

Finger flexor spasticity was measured immediately before and after 10 s of a stimulus using the MAS. The MAS score was assessed by an occupational therapist with many years of experience using the MAS. She manually extended the participant’s fingers from a position of maximum possible flexion to maximum possible extension over a duration of about one second, performing 5 to 8 such sequential extensions. The 6-point ordinal MAS [27,29] was used to rate the degree of muscle tone: 0—no increase in muscle tone; 1—slight increase in muscle tone manifested by a catch and release or by minimal resistance at the end of the range of motion; 1+—slight increase in muscle tone manifested by a catch followed by minimal resistance throughout the remainder (less than half) of the range of motion; 2—more marked increase in muscle tone through most of the range of motion; 3—considerable increase in muscle tone, passive movement difficult; 4—affected part is rigid in flexion or extension. The therapist recorded the MAS score on a data collection form immediately after making the assessment. Blinded assessment was not feasible within this pilot design, because the stimuli and the movements they evoked could not be concealed from the assessor. Change in hypertonia was defined relative to the pre-stimulus MAS score recorded within the same trial, not relative to the participant’s enrollment baseline. A trial was classified as showing no reduction whenever the post-stimulus MAS score was equal to or higher than that trial’s own pre-stimulus score. Because the MAS is an ordinal scale whose levels describe qualitatively different patterns of resistance rather than equal increments, the post-stimulus MAS category was analyzed directly rather than as an arithmetic pre-to-post difference. No electromyographic or other electrophysiological signals were recorded.

### 2.5. Statistical Analysis

The primary hypothesis was that combining percussive FMV with electrical stimulation would result in greater reductions in post-stimulus MAS scores than percussive FMV alone; the primary outcome was the post-stimulus MAS category. To test this, multinomial logistic regression was used to compare the probabilities of four post-treatment outcomes (no change, MAS_post_ = 2, MAS_post_ = 1+, and MAS_post_ = 0–1) across 11 treatment categories. A multinomial rather than an ordinal model was used because these categories are not ordered on a common scale: “no change” is defined relative to each trial’s pre-stimulus score, and MAS_post_ = 2 cannot occur in trials that began at MAS 2. The treatment categories included single-modality treatments—percussive FMV (FMV_p_), non-percussive FMV (FMV_np_), NMES at 35 Hz (NMES35), NMES at 100 Hz (NMES100), and TENS—and combinations of two modalities: FMV_p_ + NMES35, FMV_p_ + NMES100, FMV_p_ + TENS, NMES35 + NMES100, NMES35 + TENS, and NMES100 + TENS (Table 1). FMV_p_ was chosen as the baseline treatment due to its previously demonstrated effects on spasticity [17].

Because the study included repeated measurements, participant was included in the model as a fixed-effect covariate so that treatment effects were estimated from within-participant contrasts; with only five participants, the between-participant variance of a mixed-effects model could not be estimated reliably. As a sensitivity analysis, standard errors were also clustered by session (31 sessions). Results are reported as average marginal effects (i.e., the difference in the predicted probability of each outcome between a treatment category and FMV_p_ averaged over all trials) with 95% confidence intervals (CI) as measures of effect size. To check whether any single participant drove the pooled estimates, the model was refitted leaving each participant out in turn, and outcomes were also summarized for each participant (Section 3.4).

Pairwise comparisons between FMV_p_ and the other 10 treatment categories were the primary comparisons and were performed using Z-tests for differences in predicted probabilities, with a significance threshold of 0.05; we also indicate which differences remain significant after Bonferroni correction for the 10 comparisons within each outcome (α = 0.005) and for all 40 comparisons (α = 0.00125). The impact of treatment side (both, spastic, and antagonist) and FMV position (tendon or muscle belly) was evaluated in secondary, exploratory analyses using proportional odds logistic regression with participant and treatment category as covariates. Likelihood-ratio tests of nested models (analysis of variance, ANOVA) were used to assess whether the inclusion of participant, treatment category, side, or position significantly improved model fit. All statistical analyses were performed in Python 3.11 (pandas 3.0, SciPy 1.17, statsmodels 0.15).

The number of trials per condition (Table 1; Supplementary Table S1) reflects the repetitions completed: some conditions were repeated more than the planned three times, one participant did not complete two groupings, and one completed only three sessions (Section 3.1). No trials were excluded and no data were imputed. To assess bias from repetition or incomplete recovery to baseline, a restricted dataset was defined comprising the first three repetitions of each stimulus condition per participant, retaining only trials with a pre-stimulus MAS of 3. Because estimates from the restricted and full datasets agreed closely (Section 3.2), the full dataset, which uses all available trials, was used for all analyses.

## 3. Results

### 3.1. Participant Characteristics

Five participants were enrolled in the study (Table 3). Their average (standard deviation) age was 49 (12) years, and their time post-stroke ranged from 8 months to 16 years. The average (SD) UEFM score was 21 (4.9), with three participants scoring ≤19, indicative of severe upper limb impairment [30]. A total of 804 trials were conducted, including 107 control non-intervention trials, and 697 intervention trials (Table 1). These trials are repeated measurements from five individuals and not independent observations: four participants completed seven sessions each (176–186 trials per participant), whereas one participant (FMV05) completed three sessions (79 trials) in which each condition was tested once or twice; one participant (FMV02) did not complete the NMES35 + TENS and NMES35 + NMES100 groupings. The number of trials contributed by each participant to each condition is given in Supplementary Table S1. The pre-stimulus MAS score was 3 in 91.3% of trials and 2 in the remaining 8.7%. No adverse events were observed or spontaneously reported: there were no skin reactions at the electrode or applicator sites, no reports of pain during stimulation (intensities were set below the pain threshold by design), no unintended movements beyond the expected stimulation-evoked finger flexion or extension, and no participant withdrew or discontinued a session because of discomfort.

### 3.2. Sensitivity Analysis for Bias

To address potential bias from trial repetition or incomplete recovery to baseline, a Pearson’s Chi-squared test with Yates’ correction was used to evaluate (1) the independence of post-stimulus outcomes and trial repetition (more than three times) and (2) the relationship between pre-stimulus MAS scores and reductions post-stimulus. Neither test indicated bias: the proportion of intervention trials showing any reduction in MAS did not differ between the first three repetitions of a condition and later repetitions (84.3% vs. 88.6%; χ^2^ = 0.70, *p* = 0.40) or between trials with a pre-stimulus MAS of 3 and of 2 (84.9% vs. 83.3%; χ^2^ = 0.02, *p* = 0.89). Sensitivity analysis was conducted on a restricted dataset comprising the first three trials of each condition with pre-stimulus MAS = 3. The estimated probability differences relative to FMV_p_ obtained from this subset (569 intervention trials) were highly correlated (r = 0.976) with those from the full dataset (697 trials). In the restricted dataset, the increase in MAS_post_ = 0–1 with NMES100 + FMV_p_ remained significant (20.2%, 95% CI: 5.5–34.8%), whereas that with NMES35 + FMV_p_ was attenuated (14.3%, 95% CI: −0.3 to 28.8%). The full dataset was therefore used for all subsequent analyses because it maximizes the number of trials contributing to each estimate.

### 3.3. Immediate Post-Stimulus Changes in MAS Score

In the control condition (no intervention), no reduction in post-stimulus MAS score occurred in 90 of 107 trials (84.1%), indicating that hypertonia was generally stable in the absence of stimulation. In the remaining 17 control trials (FMV01-FMV05: 5, 4, 2, 5, and 1), the MAS most often fell by one grade, from 3 to 2 (12 trials). By contrast, spasticity was reduced in 84.8% of the 697 intervention trials. Likelihood-ratio (ANOVA) tests of nested models demonstrated that both the participant (*p* = 5.99 × 10^−12^) and the treatment category (*p* = 3.07 × 10^−15^) were significant predictors of post-stimulus outcomes, indicating that treatment modality and individual variability strongly influenced spasticity reduction; participant was retained as a covariate in all models. In the secondary analyses, no significant differences in post-stimulus MAS scores were observed between treatment sides (both, spastic, and antagonist, *p* = 0.87) or between FMV positions (tendon and muscle belly, *p* = 0.86).

**Figure 2:**
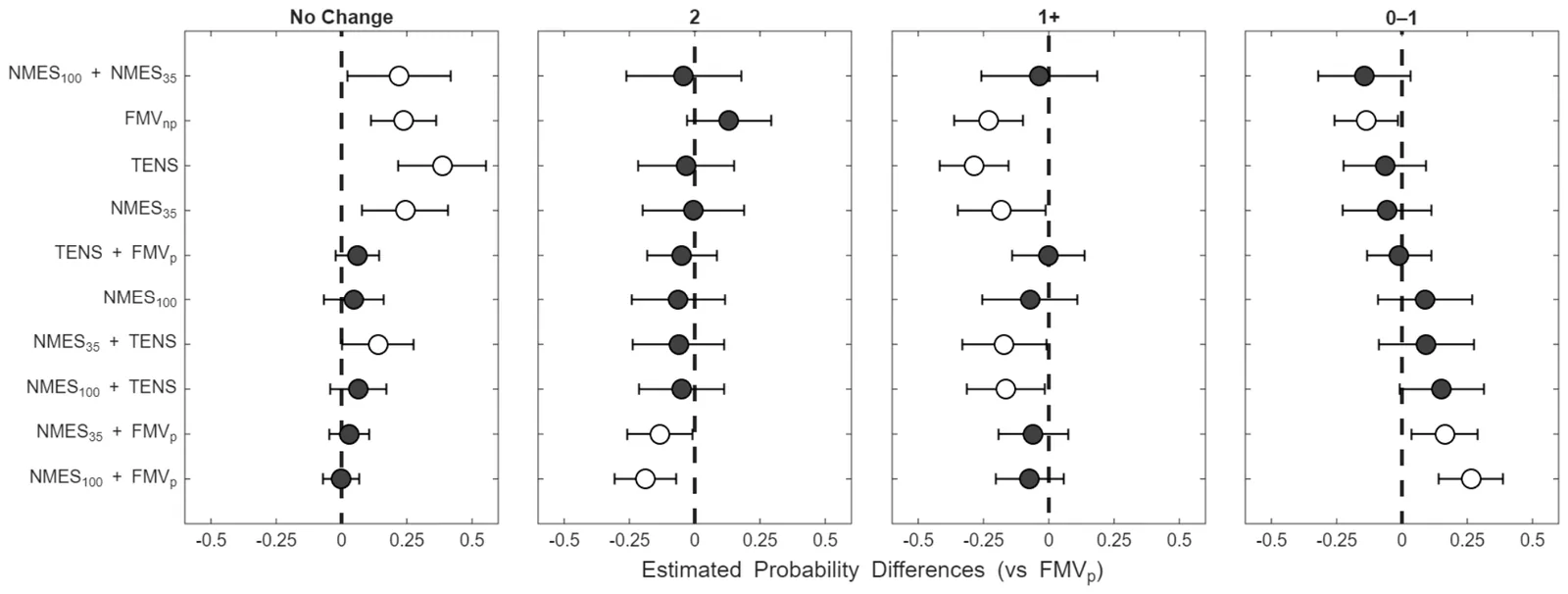
Estimated Probability Differences for Post-Stimulus MAS Outcomes Across Treatment Conditions. Estimates are average marginal effects from the multinomial logistic regression model adjusted for participant. Each panel represents a distinct post-stimulus MAS outcome: No Change, MAS_post_ = 2, MAS_post_ = 1+, and MAS_post_ = 0–1. The vertical dashed line indicates the reference condition of percussive FMV (FMV_p_), serving as the baseline for comparison. Points to the right of the dashed line indicate a higher probability of achieving the specified outcome compared to FMV_p_ alone. Error bars represent 95% confidence intervals, with open white markers indicating statistically significant differences from FMV_p_ (*p* < 0.05).

Figure 2 illustrates the model-estimated probability differences (average marginal effects adjusted for participant) for achieving specific post-stimulus MAS outcomes across treatment conditions, with each panel representing a distinct outcome. FMV_p_ combined with NMES showed the most favorable outcomes. For NMES35 + FMV_p_, the probability of achieving MAS_post_ = 0–1 (Figure 2, fourth panel) was 16.3% (95% CI: 3.5–29.0%) higher than FMV_p_ alone, and for NMES100 + FMV_p_, the increase was even greater at 26.4% (95% CI: 13.9–38.8%). These combinations also reduced the probability of achieving MAS_post_ = 2 (Figure 2, second panel), with NMES35 + FMV_p_ reducing it by 13.4% (95%CI: 0.8–25.9%) and NMES100 + FMV_p_ by 18.8% (95% CI: 6.9–30.7%).

In contrast, FMV_np_ and TENS alone reduced MAS less often than FMV_p_. FMV_np_ increased the likelihood of no reduction in MAS score (Figure 2, first panel) by 23.7% (95% CI: 11.3–36.1%) and reduced the probability of achieving MAS_post_ = 0–1 (Figure 2, fourth panel) by 13.8% (95% CI: 1.6–26.0%). TENS alone increased the likelihood of no reduction by 38.5% (95% CI: 21.7–55.4%). Similarly, treatments such as TENS + FMV_p_, NMES100 + TENS, and NMES100 alone showed no significant differences in the probability of no reduction or of MAS_post_ = 0–1 compared to FMV_p_, suggesting limited added benefit. Under a Bonferroni correction for the 10 comparisons within each outcome (α = 0.005), the higher probability of MAS_post_ = 0–1 (*p* < 0.001) and the lower probability of MAS_post_ = 2 (*p* = 0.002) with NMES100 + FMV_p_, the higher probability of no reduction with TENS (*p* < 0.001), FMV_np_ (*p* < 0.001), and NMES35 alone (*p* = 0.004), and the lower probability of MAS_post_ = 1+ with TENS (*p* < 0.001) and FMV_np_ (*p* < 0.001) remained significant. The differences associated with NMES35 + FMV_p_ (*p* = 0.012 and *p* = 0.037) and the lower probability of MAS_post_ = 0–1 with FMV_np_ (*p* = 0.027) did not survive correction and should be regarded as exploratory. The NMES100 + FMV_p_ effect on MAS_post_ = 0–1 also survived correction for all 40 comparisons, and remained significant with session-clustered standard errors (95% CI: 13.4–39.3%, *p* < 0.001); the NMES35 + FMV_p_ differences did not (*p* = 0.07). Overall, these findings suggest that in this sample, FMV_p_ combined with NMES, particularly at 100 Hz, produced larger immediate reductions in MAS than the other tested conditions.

### 3.4. Individual Participant Responses

**Figure 3:**
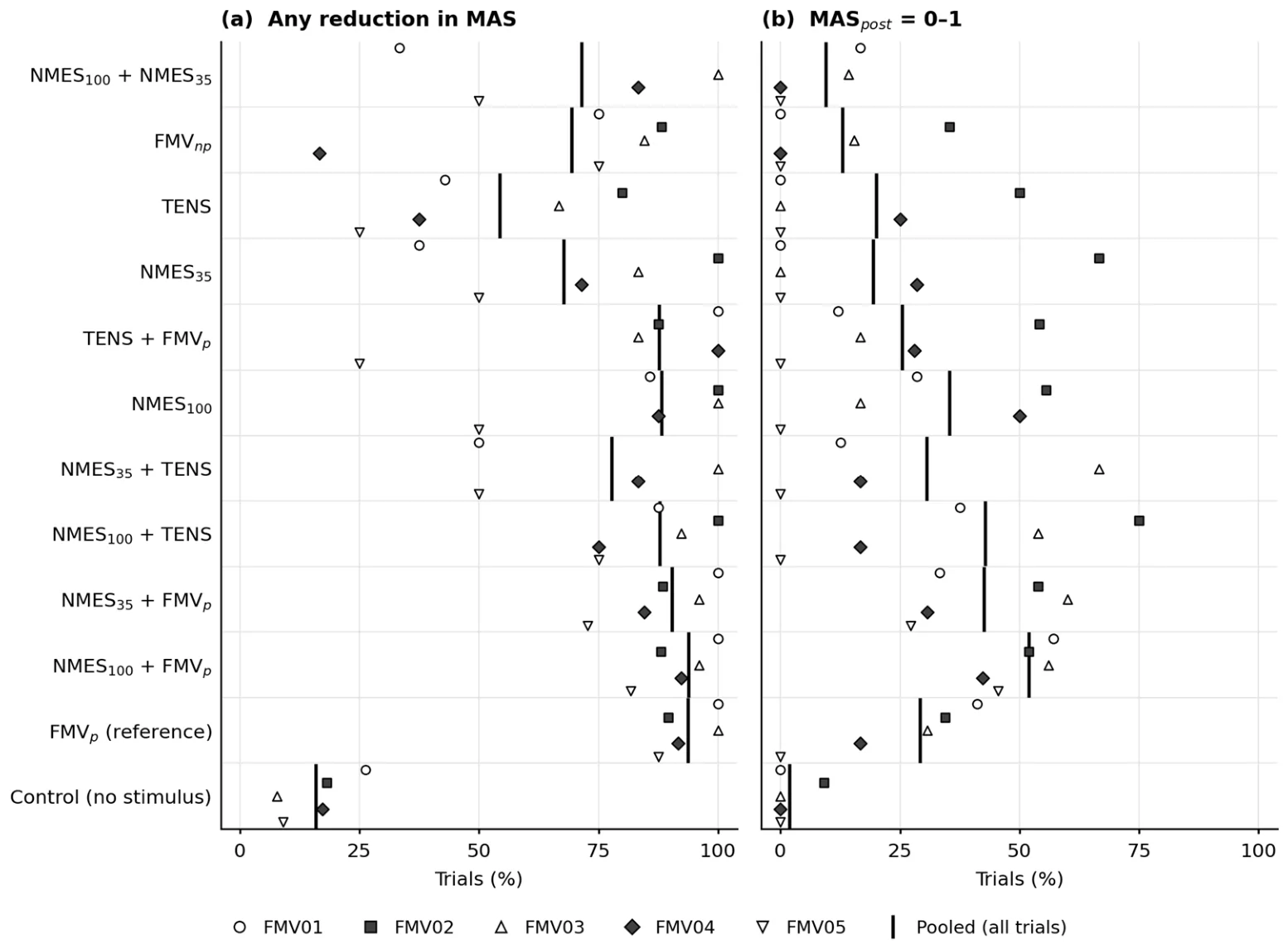
Individual participant responses by treatment category: percentage of trials in which (**a**) the MAS decreased by at least one grade and (**b**) the post-stimulus MAS was 0–1. Symbols show individual participants; vertical lines show the percentage of all trials in that category, pooled across participants. Categories are ordered as in Figure 2, with the FMV_p_ reference and control conditions at the bottom.

Outcomes were also examined for each participant (Figure 3; Supplementary Table S2). NMES100 + FMV_p_ produced MAS_post_ = 0–1 more often than FMV_p_ alone in every participant (by 16–45 percentage points; exact sign test, one-sided *p* = 0.031), and NMES35 + FMV_p_ did so in four of five. FMV_np_ and TENS produced a reduction in MAS less often than FMV_p_ in all five participants, although responses to these conditions varied widely (e.g., FMV_np_ reduced MAS in 2 of 12 trials for FMV04 but 15 of 17 for FMV02). Omitting each participant in turn changed the modeled difference between NMES100 + FMV_p_ and FMV_p_ for MAS_post_ = 0–1 only modestly (24.5–27.0%, vs. 26.4% with all participants); the corresponding NMES35 + FMV_p_ estimate varied more (12.3–22.8%). Overall responsiveness also differed between participants, consistent with the significant participant term: the proportion of intervention trials ending at MAS_post_ = 0–1 ranged from 12% (FMV05) to 50% (FMV02).

## 4. Discussion

This exploratory study investigated the immediate effects of various neuromodulation techniques on finger flexor hypertonia, as assessed by the MAS after 10-s applications, in repeated trials in five stroke survivors. The findings indicate that among the different interventions tested, the combination of percussive focal muscle vibration with neuromuscular electrical stimulation showed the largest shift toward lower MAS scores (Figure 2 and Figure 3), suggesting a larger immediate effect on hypertonia. Specifically, this combined approach demonstrated a higher probability of a post-stimulus MAS of 0–1 compared to other stimulation methods. In addition to increasing the likelihood of MAS_post_ = 0–1, these combinations also reduced the probability of reducing spasticity from MAS_pre_ = 3 to MAS_post_ = 2, a moderate reduction in spasticity. This finding may be clinically relevant because while MAS_post_ = 2 reflects moderate reductions in spasticity, further reductions to MAS_post_ = 1+ or MAS_post_ = 0–1 may contribute to improved motor control and functional performance, as these outcomes are associated with lower muscle tone and increased movement efficiency. By lowering the probability of this intermediate outcome, combined FMV_p_ and NMES stimulation appears to shift the distribution of outcomes toward more favorable results, indicating that this combination merits further evaluation.

Other conditions, including NMES or TENS applied alone, non-percussive FMV, and the combinations of NMES with TENS or of the two NMES frequencies, showed limited effectiveness in achieving comparable spasticity reductions. These results suggest that percussive FMV combined with NMES produces larger immediate reductions in MAS than the other conditions tested. Such a combined effect is plausible, as FMV and NMES engage partly different inhibitory pathways that may act in concert.

The most likely mechanism by which FMV may immediately reduce spasticity is by generating Ia input to the spinal cord through activation of the primary endings of muscle spindles [20]. In humans, these spindle endings respond to vibration frequencies up to 100 Hz, with FMV frequencies of 80–100 Hz effectively modulating motor potentials via spinal or cortical mechanisms [20,21,22]. Ia activity from the antagonist muscle increases inhibitory input to alpha motor neurons of the spastic muscle through reciprocal inhibition, reducing excitability. When FMV is applied to the spastic muscle, it can also elicit a tonic vibration reflex that further inhibits alpha motor neuron excitability via Renshaw cells, contributing to sustained spasticity reduction [31]. Additionally, FMV-generated Ia afferent activity reaches the primary somatosensory and motor cortices, affecting corticospinal excitability [23,24,25]. FMV may also activate Golgi tendon organs, enhancing inhibition, particularly when the muscle is contracted [20]. In this study, percussive FMV produced immediate reductions in hypertonia more often than non-percussive FMV. One possible explanation is larger displacement amplitude (5 mm as compared to 0.01 to 2.0 mm in previous studies on FMV [32]), which may activate muscle spindles more strongly, whereas the smaller amplitude of non-percussive FMV may act primarily on skin receptors. This interpretation remains speculative, because the displacement amplitude of the non-percussive device and the contact force of either device were not measured. The two devices also differed in vibration frequency (60 vs. 100 Hz), applicator size and shape, and the sensation they produced, and any of these could contribute to the difference.

Interestingly, no significant difference in outcomes was observed between FMV applied to the muscle belly and the tendon in our study, suggesting that both locations are similarly effective in activating Ia afferents to reduce spasticity. This contrasts with recent findings that reported differences in effectiveness between these application sites, potentially due to differences in study protocols [33]. Specifically, the prior study fixed participants’ wrist joints in maximal extension during vibration, which potentially increased muscle spindle stretch and amplified Ia afferent input. In our protocol, participants’ joints were not fixed in this manner, which could have limited the extent of vibration-induced spindle activation and contributed to the observed lack of differentiation between application sites. While vibrating the muscle belly is thought to preferentially activate Ia afferents and vibrating the tendon activates Ib afferents, both pathways ultimately reduce alpha motor neuron excitability, which may explain the similar effectiveness observed in our study. Further research is warranted to explore how application site, vibration parameters, and joint mechanics influence these outcomes.

The limited effect of TENS was similar to non-percussive FMV and less pronounced than NMES, likely due to TENS’s inability to produce muscle contractions or effectively activate muscle spindles. In contrast, both 35 Hz and 100 Hz NMES applied in this study involved current intensities that recruited motor axons, resulting in muscle contractions and indirect activation of Ia afferents via spindle stretching. NMES may reduce spasticity by facilitating Ia reciprocal inhibition, Ib activation via Renshaw cell recurrent inhibition, or cutaneous sensory activation [18]. While NMES has shown variable effects on spasticity, including mixed results in previous studies [34,35,36,37], this study aligns with meta-analytic findings that NMES combined with other therapies (e.g., exercise, botulinum toxin injections, conventional therapy) yields better outcomes than NMES alone [18]. Although none of the studies combined NMES with FMV, the observation that NMES used alongside other interventions achieved better outcomes than NMES alone aligns with our finding that NMES paired with FMV produced larger immediate reductions in MAS than FMV alone.

This study has several limitations that should be considered when interpreting the results. First, the assessing occupational therapist was not blinded to the condition being applied, nor were the statistical analysts, and the MAS is a subjective ordinal measure dependent on clinician judgment with moderate inter-rater reliability [27,29]; together these may introduce measurement and expectancy bias, which would tend to favor the conditions the assessor expected to work. The embedded control condition offers partial reassurance, in that no reduction in MAS occurred in 84.1% of control trials, but it does not exclude expectancy effects. Second, the MAS was the sole outcome measure. Its ordinal levels cannot resolve differences smaller than one grade, and no quantitative measure of resistance to passive stretch, joint torque, or muscle activity was obtained, so the magnitude and mechanism of the observed changes remain uncharacterized. Future studies should therefore pair the MAS with a blinded rater and with objective measures of spasticity or muscle activity, such as resistance torque during standardized passive stretch, the Tardieu scale, or surface electromyography of the finger flexors. Third, although the protocol was designed to minimize carryover through a voluntary maximal contraction at the start of each trial, at least one minute of rest between trials, and periodic control trials, the order in which trials were conducted within each session was not entered into the study dataset, so carryover and order effects could not be tested. Future studies should record trial sequence and test for these effects. The order of conditions was also determined by the investigators at the time of testing rather than from a prospectively generated schedule, so an unconscious bias in ordering cannot be excluded. Because conditions were tested in blocks by grouping, and groupings correspond to the treatment categories analyzed, time-in-session and session effects could be partly confounded with treatment. The maximal fist contraction that began each trial was intended to evoke finger flexor hypertonia and reset carryover, but when repeated over a session it could itself induce fatigue or alter muscle tone, changing the MAS independently of the stimulus. The stability of the MAS in the control trials, which included the same contraction (no reduction in 84.1%), and the absence of a difference between early and later repetitions of a condition (Section 3.2) provide partial reassurance that fatigue and carry-over did not systematically distort the results. Fourth, the sample comprised only five participants, who differed in age, time since stroke, paretic side, and impairment (Table 3), and in their overall responsiveness (Section 3.4). Because participant was modeled as a fixed effect, the results describe these five participants, and the observed statistical significance does not establish generalizability, even though the advantage of NMES100 + FMV_p_ over FMV_p_ was seen in every participant. Unequal numbers of trials across conditions and participants (Supplementary Table S1) further reduced precision, and replication in larger cohorts is needed to confirm these results. Fifth, each stimulus was applied for only 10 s, considerably shorter than application times that would likely be necessary therapeutically. The ranking of conditions observed here may not hold at longer durations, since a modality that acts quickly is not necessarily the one that produces the largest or most durable effect after several minutes of application. In addition, neither FMV contact force nor the displacement magnitude of the non-percussive device were measured. Sixth, hypertonia was reassessed immediately after each stimulus, so these data establish only that certain combinations produce an immediate reduction in MAS and say nothing about how long that reduction persists. Because clinical benefit depends on whether reduced tone lasts long enough to permit stretching, splinting, hygiene, or task practice, characterizing the time course of the effect is a necessary next step before combined percussive FMV and NMES could be recommended for therapeutic use. Seventh, with 48 conditions and 40 pairwise comparisons, the study was exploratory and hypothesis-generating; only the comparisons that survived Bonferroni correction (Section 3.3) should be considered reasonably robust, and the remainder require confirmation. Finally, the conditions tested did not include NMES applied to opposing muscles without concurrent FMV, which remains unexplored and could provide insight into alternative methods for spasticity management.

Within the limits described above, the results showed that percussive FMV produced immediate reductions in MAS more often than non-percussive FMV, and that combining percussive FMV with NMES produced a larger immediate reduction in MAS than percussive FMV alone. Currently, no commercially available device integrates percussive FMV and NMES into a single unit, highlighting a gap that must be addressed to advance future research. Developing such a device would enable more comprehensive investigations into combined spasticity treatments and facilitate user-controlled management during daily activities. Advances in miniaturized vibration motors could make the development of such a wearable device feasible, empowering patients or caregivers to administer therapy independently, thus extending treatment duration beyond clinical visits. Additionally, a wearable device could facilitate long-term studies on the effects of extended or frequent FMV + NMES application, providing valuable data on its sustained efficacy and safety in stroke survivors or other populations affected by spasticity. Before such development is warranted, however, the present findings need to be confirmed in a study designed for that purpose: a larger and more homogeneous sample; a prospectively defined, randomized, and recorded order of conditions; blinded assessment of the MAS; objective measures of spasticity and muscle activity; a factorial design that permits a formal test of the FMV × NMES interaction; longer stimulation durations; and follow-up assessments at fixed intervals after stimulation to determine how long any reduction persists.

## 5. Conclusions

In this exploratory pilot study of five stroke survivors, 10-s applications of percussive FMV combined with NMES, particularly at 100 Hz, produced the largest immediate reductions in finger flexor MAS scores among the conditions tested, and the 100 Hz combination brought MAS to 0–1 more often than percussive FMV alone in every participant. These findings provide preliminary evidence of an immediate reduction in hypertonia under the tested conditions. Larger studies with a randomized and recorded condition order, blinded and objective outcome assessment, longer stimulation periods, and follow-up are now needed to establish the duration and clinical relevance of this effect before combined percussive FMV and NMES is developed for therapeutic use.

## Figures and Tables

**Table 1:** Test Conditions, grouped by stimulus modality or pair of modalities (analysis category).

Test Condition	Stimulus Applied to Flexor (Spastic) Side	Stimulus Applied to Extensor (Antagonist) Side	Trials Analyzed (Restricted/Full Dataset)
1 (control)	None	None	97/107
Percussive FMV (FMV_p_) Only			
2	FMV_p_ (M)	None	14/20
3	FMV_p_ (T)	None	13/18
4	None	FMV_p_ (M)	14/22
5	None	FMV_p_ (T)	13/19
Non-Percussive FMV (FMV_np_) Only			
6	FMV_np_ (M)	None	14/15
7	FMV_np_ (T)	None	14/15
8	None	FMV_np_ (M)	14/17
9	None	FMV_np_ (T)	14/15
TENS Only			
10	TENS	None	12/17
11	None	TENS	13/18
TENS + FMV_p_			
12	FMV_p_ (M)	TENS	13/13
13	FMV_p_ (T)	TENS	12/13
14	TENS	FMV_p_ (M)	13/13
15	TENS	FMV_p_ (T)	13/13
16	FMV_p_ (M) + TENS	None	12/13
17	FMV_p_ (T) + TENS	None	12/13
18	None	FMV_p_ (M) + TENS	11/14
19	None	FMV_p_ (T) + TENS	11/14
NMES35 Only			
20	NMES35	None	13/16
21	None	NMES35	13/15
NMES35 + FMV_p_			
22	FMV_p_ (M)	NMES35	11/14
23	FMV_p_ (T)	NMES35	10/14
24	NMES35	FMV_p_ (M)	12/15
25	NMES35	FMV_p_ (T)	12/14
26	FMV_p_ (M) + NMES35	None	11/13
27	FMV_p_ (T) + NMES35	None	12/14
28	None	FMV_p_ (M) + NMES35	13/17
29	None	FMV_p_ (T) + NMES35	13/14
NMES100 Only			
30	NMES100	None	13/16
31	None	NMES100	13/18
NMES100 + FMV_p_			
32	FMV_p_ (M)	NMES100	12/14
33	FMV_p_ (T)	NMES100	11/13
34	NMES100	FMV_p_ (M)	12/13
35	NMES100	FMV_p_ (T)	11/13
36	FMV_p_ (M) + NMES100	None	11/15
37	FMV_p_ (T) + NMES100	None	13/15
38	None	FMV_p_ (M) + NMES100	13/27
39	None	FMV_p_ (T) + NMES100	14/19
NMES35 + TENS			
40	NMES35	TENS	7/9
41	TENS	NMES35	7/9
42	NMES35 + TENS	None	8/9
43	None	NMES35 + TENS	8/9
NMES35 + NMES100			
44	NMES35	NMES100	9/10
45	NMES100	NMES35	9/11
NMES100 + TENS			
46	NMES100	TENS	12/12
47	TENS	NMES100	12/12
48	NMES100 + TENS	None	10/12
49	None	NMES100 + TENS	12/13

Each numbered row is one test condition. The second and third columns give the stimulus applied to the flexor (spastic) side and to the extensor (antagonist) side of the forearm, respectively; for FMV, (M) and (T) denote application to the muscle belly or to the tendon (Figure 1). Shaded rows name the condition grouping, which is also the treatment category used in the statistical analysis (Section 2.5). The last column gives the number of trials in the restricted dataset (first three repetitions of each stimulus condition per participant, retaining only trials with pre-stimulus MAS = 3; all control trials with pre-stimulus MAS = 3) and in the full dataset (all trials with recorded pre- and post-stimulus MAS), summed over participants. FMV_p_, percussive focal muscle vibration; FMV_np_, non-percussive focal muscle vibration; NMES35 and NMES100, neuromuscular electrical stimulation at 35 Hz and 100 Hz; TENS, transcutaneous electrical nerve stimulation.

**Table 2:** Sequence of events in each experimental trial.

Step	Event	Instruction or Action	Duration
1	Voluntary contraction	“Make a fist. Go! Go! Go!” Evokes the participant’s maximal finger flexor hypertonia and resets any carry-over from the preceding trial.	≈3 s
2	Relaxation	“Now relax your hand.”	not timed
3	Pre-stimulus assessment	Occupational therapist rates finger flexor tone with the MAS (5–8 passive finger extensions of ≈1 s each) and records the score.	not timed
4	Stimulus	Investigator applies FMV, ES, or their combination (Table 1) with the participant relaxed and neither aiding nor resisting evoked movement. ES ramps on over 1 s and shuts off automatically; FMV is timed with a countdown timer.	10 s
5	Post-stimulus assessment	Same MAS procedure, begun immediately after the stimulus ends; participant reports whether the hand feels tighter, looser, or unchanged.	not timed
6	Rest	Minimum rest before the next trial begins.	≥1 min
—	Control trial	Steps 1–3, 10 s without any stimulus, then steps 5–6; performed after every 8 stimulus trials. If the MAS fell, several minutes of rest were given and the control trial repeated; the session was ended if reductions persisted.	as above

**Table 3:** Participant Demographics and Clinical Characteristics.

Participant	Sex	Age (y)	Time Post-Stroke (mo)	Paretic Side (R or L)	Baseline UEFM (Max 66)	Baseline Finger Flexor MAS (Max 4)
FMV01	F	34	8	R	18	3
FMV02	M	61	192	R	23	3
FMV03	M	60	13	L	19	3
FMV04	M	51	25	L	29	3
FMV05	M	38	56	R	17	3

## Data Availability

The data supporting the findings of this study are available from the corresponding authors upon reasonable request. Due to privacy and ethical restrictions, the data are not publicly deposited.

## Supplementary Materials

The following supporting information can be downloaded at: https://www.mdpi.com/article/10.3390/bioengineering13101228/s1, Table S1: Number of trials contributed by each participant to each test condition; Table S2: Individual participant responses by treatment category.

## Abbreviations

The following abbreviations are used in this manuscript:

FMV	Focal muscle vibration
NMES	Neuromuscular electrical stimulation
TENS	Transcutaneous electrical nerve stimulation
MAS	Modified Ashworth Scale
RF	Reticular formation
dRST	Dorsal reticulospinal tract
ES	Electrical stimulation
UEFM	Upper extremity Fugl-Meyer
ANOVA	Analysis of variance
CI	Confidence interval
SD	Standard deviation
EDC	Extensor digitorum communis
FDS	Flexor digitorum superficialis
