# Supplementary material for: Focal Muscle Vibration Combined with Electrical Stimulation for Post-Stroke Upper Limb Spasticity: A Pilot Study
Source: Bioengineering (Basel). 2026 Oct 21;13(10):1228. doi: 10.3390/bioengineering13101228 (PMC13701424; doi:10.3390/bioengineering13101228)
Supplement: Supplementary file 1 [file bioengineering-13-01228-s001.zip › bioengineering-4566504-supplementary.pdf]

bioengineering

MDPI

Supplementary Materials

Focal Muscle Vibration Combined with Electrical Stimulation for Post-Stroke Upper Limb Spasticity: A Pilot Study

Christopher L. Pulliam [1,*], Terri Z. Hisel [2], Sarah E. Grabinski [3], Victoria C. Whitehair [2,4] and Jayme S. Knutson [2,4]

[1] Department of Biomedical Engineering, Case Western Reserve University, Cleveland, OH, 44106, USA
[2] Department of Physical Medicine and Rehabilitation, MetroHealth Rehabilitation Institute, Cleveland, OH, 44109, USA
[3] Department of Population and Quantitative Health Sciences, Case Western Reserve University, Cleveland, OH, 44106, USA;
[4] Department of Physical Medicine and Rehabilitation, Case Western Reserve University, School of Medicine, Cleveland, OH, 44106, USA
* Correspondence: pulliam@case.edu

Table S1. Number of trials contributed by each participant to each test condition, given as full dataset / restricted dataset. Condition numbers and the stimuli applied to each side of the forearm correspond to Table 2 of the main text; (M) and (T) denote FMV applied to the muscle belly or the tendon. The full dataset comprises all trials with recorded pre- and post-stimulus MAS scores; the restricted dataset comprises, for each participant and condition, the first three repetitions, retaining only trials with a pre-stimulus MAS of 3. FMV02 did not complete conditions 40–45 (the NMES35 + TENS and NMES35 + NMES100 groupings); FMV05 completed three sessions in which each condition was tested once or twice. FMV_p, percussive focal muscle vibration; FMV_{np}, non-percussive focal muscle vibration; NMES35 and NMES100, neuromuscular electrical stimulation at 35 Hz and 100 Hz; TENS, transcutaneous electrical nerve stimulation.

Condition	Flexor (spastic) side	Extensor (antagonist) side	FMV01	FMV02	FMV03	FMV04	FMV05	All participants
1	None	None	19 / 16	22 / 19	26 / 23	29 / 28	11 / 11	107 / 97
2	FMV_p (M)	None	5 / 3	7 / 3	3 / 3	3 / 3	2 / 2	20 / 14
3	FMV_p (T)	None	3 / 3	7 / 3	3 / 2	3 / 3	2 / 2	18 / 13
4	None	FMV_p (M)	5 / 3	8 / 3	4 / 3	3 / 3	2 / 2	22 / 14
5	None	FMV_p (T)	4 / 3	7 / 3	3 / 2	3 / 3	2 / 2	19 / 13
6	FMV_{np} (M)	None	3 / 3	4 / 3	3 / 3	3 / 3	2 / 2	15 / 14
7	FMV_{np} (T)	None	3 / 3	4 / 3	3 / 3	3 / 3	2 / 2	15 / 14
8	None	FMV_{np} (M)	3 / 3	5 / 3	4 / 3	3 / 3	2 / 2	17 / 14
9	None	FMV_{np} (T)	3 / 3	4 / 3	3 / 3	3 / 3	2 / 2	15 / 14
10	TENS	None	3 / 3	5 / 3	3 / 2	4 / 2	2 / 2	17 / 12
11	None	TENS	4 / 3	5 / 3	3 / 3	4 / 2	2 / 2	18 / 13
12	FMV_p (M)	TENS	3 / 3	3 / 3	3 / 3	3 / 3	1 / 1	13 / 13
13	FMV_p (T)	TENS	3 / 3	3 / 3	3 / 2	3 / 3	1 / 1	13 / 12
14	TENS	FMV_p (M)	3 / 3	3 / 3	3 / 3	3 / 3	1 / 1	13 / 13
15	TENS	FMV_p (T)	3 / 3	3 / 3	3 / 3	3 / 3	1 / 1	13 / 13
16	FMV_p (M) + TENS	None	3 / 3	3 / 3	3 / 2	3 / 3	1 / 1	13 / 12
17	FMV_p (T) + TENS	None	3 / 3	3 / 3	3 / 2	3 / 3	1 / 1	13 / 12
18	None	FMV_p (M) + TENS	4 / 3	3 / 2	3 / 2	3 / 3	1 / 1	14 / 11
19	None	FMV_p (T) + TENS	3 / 3	3 / 3	3 / 2	4 / 2	1 / 1	14 / 11

Condition	Flexor (spastic) side	Extensor (antagonist) side	FMV01	FMV02	FMV03	FMV04	FMV05	All participants
20	NMES35	None	4 / 3	3 / 3	3 / 3	4 / 2	2 / 2	16 / 13
21	None	NMES35	4 / 3	3 / 2	3 / 3	3 / 3	2 / 2	15 / 13
22	FMV_P (M)	NMES35	3 / 3	3 / 3	3 / 2	4 / 2	1 / 1	14 / 11
23	FMV_P (T)	NMES35	3 / 3	3 / 2	3 / 2	4 / 2	1 / 1	14 / 10
24	NMES35	FMV_P (M)	3 / 3	4 / 3	4 / 2	3 / 3	1 / 1	15 / 12
25	NMES35	FMV_P (T)	3 / 3	4 / 3	3 / 2	3 / 3	1 / 1	14 / 12
26	FMV_P (M) + NMES35	None	3 / 3	3 / 2	3 / 2	3 / 3	1 / 1	13 / 11
27	FMV_P (T) + NMES35	None	3 / 3	3 / 2	3 / 2	3 / 3	2 / 2	14 / 12
28	None	FMV_P (M) + NMES35	6 / 3	3 / 3	3 / 2	3 / 3	2 / 2	17 / 13
29	None	FMV_P (T) + NMES35	3 / 3	3 / 2	3 / 3	3 / 3	2 / 2	14 / 13
30	NMES100	None	3 / 3	4 / 3	3 / 3	4 / 2	2 / 2	16 / 13
31	None	NMES100	4 / 3	5 / 3	3 / 3	4 / 2	2 / 2	18 / 13
32	FMV_P (M)	NMES100	3 / 3	3 / 3	3 / 2	4 / 3	1 / 1	14 / 12
33	FMV_P (T)	NMES100	3 / 3	3 / 2	3 / 2	3 / 3	1 / 1	13 / 11
34	NMES100	FMV_P (M)	3 / 3	3 / 3	3 / 2	3 / 3	1 / 1	13 / 12
35	NMES100	FMV_P (T)	3 / 3	3 / 2	3 / 2	3 / 3	1 / 1	13 / 11
36	FMV_P (M) + NMES100	None	5 / 3	3 / 2	3 / 2	3 / 3	1 / 1	15 / 11
37	FMV_P (T) + NMES100	None	4 / 3	3 / 3	3 / 2	3 / 3	2 / 2	15 / 13
38	None	FMV_P (M) + NMES100	14 / 3	3 / 3	4 / 2	4 / 3	2 / 2	27 / 13
39	None	FMV_P (T) + NMES100	7 / 3	4 / 3	3 / 3	3 / 3	2 / 2	19 / 14
40	NMES35	TENS	2 / 1	0 / 0	3 / 2	3 / 3	1 / 1	9 / 7
41	TENS	NMES35	2 / 1	0 / 0	3 / 2	3 / 3	1 / 1	9 / 7
42	NMES35 + TENS	None	2 / 1	0 / 0	3 / 3	3 / 3	1 / 1	9 / 8
43	None	NMES35 + TENS	2 / 2	0 / 0	3 / 2	3 / 3	1 / 1	9 / 8
44	NMES35	NMES100	3 / 2	0 / 0	3 / 3	3 / 3	1 / 1	10 / 9
45	NMES100	NMES35	3 / 2	0 / 0	4 / 3	3 / 3	1 / 1	11 / 9
46	NMES100	TENS	2 / 2	3 / 3	3 / 3	3 / 3	1 / 1	12 / 12
47	TENS	NMES100	2 / 2	3 / 3	3 / 3	3 / 3	1 / 1	12 / 12
48	NMES100 + TENS	None	2 / 2	3 / 2	3 / 2	3 / 3	1 / 1	12 / 10
49	None	NMES100 + TENS	2 / 2	3 / 3	4 / 3	3 / 3	1 / 1	13 / 12
**All**			**186 / 147**	**180 / 135**	**176 / 141**	**183 / 164**	**79 / 79**	**804 / 666**

Table S2. Individual participant responses by treatment category. Each cell gives the number of trials with the specified outcome / the total number of trials for that participant and treatment category. (a) Trials in which the MAS decreased by at least one grade; (b) trials in which the post-stimulus MAS was 0–1. The "All participants" column pools the trials of all five participants; "–" indicates that the participant did not complete that grouping. The same data are plotted as percentages in Figure 3 of the main text. Abbreviations as in Table S1.

Treatment category	FMV01	FMV02	FMV03	FMV04	FMV05	All participants
**(a) Trials with any reduction in MAS: reduced / total**						
FMV_p	17/17	26/29	13/13	11/12	7/8	74/79
FMV_{np}	9/12	15/17	11/13	2/12	6/8	43/62
NMES35	3/8	6/6	5/6	5/7	2/4	21/31
NMES100	6/7	9/9	6/6	7/8	2/4	30/34
TENS	3/7	8/10	4/6	3/8	1/4	19/35
NMES35 + FMV_p	27/27	23/26	24/25	22/26	8/11	104/115
NMES100 + FMV_p	42/42	22/25	24/25	24/26	9/11	121/129
TENS + FMV_p	25/25	21/24	20/24	25/25	2/8	93/106
NMES35 + NMES100	2/6	–	7/7	5/6	1/2	15/21
NMES35 + TENS	4/8	–	12/12	10/12	2/4	28/36
NMES100 + TENS	7/8	12/12	12/13	9/12	3/4	43/49
Control (no stimulus)	5/19	4/22	2/26	5/29	1/11	17/107
**(b) Trials with MAS_{post} = 0–1: achieved / total**						
FMV_p	7/17	10/29	4/13	2/12	0/8	23/79
FMV_{np}	0/12	6/17	2/13	0/12	0/8	8/62
NMES35	0/8	4/6	0/6	2/7	0/4	6/31
NMES100	2/7	5/9	1/6	4/8	0/4	12/34
TENS	0/7	5/10	0/6	2/8	0/4	7/35
NMES35 + FMV_p	9/27	14/26	15/25	8/26	3/11	49/115
NMES100 + FMV_p	24/42	13/25	14/25	11/26	5/11	67/129
TENS + FMV_p	3/25	13/24	4/24	7/25	0/8	27/106
NMES35 + NMES100	1/6	–	1/7	0/6	0/2	2/21
NMES35 + TENS	1/8	–	8/12	2/12	0/4	11/36
NMES100 + TENS	3/8	9/12	7/13	2/12	0/4	21/49
Control (no stimulus)	0/19	2/22	0/26	0/29	0/11	2/107
